# DEPRESSION IN OPIATE ADICTION

**DOI:** 10.1192/j.eurpsy.2023.1391

**Published:** 2023-07-19

**Authors:** M. Vasiljević

**Affiliations:** Psichiatry for substance abuse, Specialist practice of psychiatry Sunce, Belgrade, Serbia

## Abstract

**Introduction:**

Miroslava Vasiljević psychiatrist employed in Specialist practice of psychiatry Sunce, Belgrade, Serbia.

**Objectives: Objectives and aims:**

Substance abuse is a major public health problem with high morbidity and mortality. Treatment – seeking opioid dependent individuals frequently report mood problems in the form of depression. The aim of this study was to evaluate the depression in patients with substance abuse.

**Methods:**

We evaluated mood problems in the form of depression and health- related quality of life (HRQoL) among patients (20) with diagnosed opiate dependance who entered detoxification program and met the criteria for abstinence in period of one month, and compared with the results of 20 healthy controls consisted of secondary medical staff.

Almost all of the patients had a total PSQI score of 6 or higher, suggestive of depression, compared with control group (PSQI < 5).Patients had problems with a depression, taking antidepressives and problem to keep up enough enthusiasm to get things done (P < 0,05), compared with the control group.

**Results:**

SF-36 scores for psysical functioning, role-physical, bodily pain, social functioning, role-emotional, and mental health were significantly lower mean compared to control group. Patients with disease had a reduced HRQoL, related to control group.

**Conclusions:**

Majority of heroin-dependent patients reported depression and reduced quality of life. These conclusions are limited, because data was collected via questionnares of patients were small. In future we plan to include more substace abuse patients.

**Disclosure of Interest:**

None Declared

